# Mental models of audit and feedback in primary care settings

**DOI:** 10.1186/s13012-018-0764-3

**Published:** 2018-05-30

**Authors:** Sylvia J. Hysong, Kristen Smitham, Richard SoRelle, Amber Amspoker, Ashley M. Hughes, Paul Haidet

**Affiliations:** 1 Michael E. DeBakey Veterans Affairs Medical Center, Houston, TX USA; 2 Baylor College of Medicine, 2002 Holcombe Blvd, Houston, TX 77030 USA; 3 VISN 4 Center for Evaluation of PACT (CEPACT), Corporal Michael J. Crescenz VA Medical Center, Philadelphia, PA USA; 4 Department of Biomedical and Health Information Sciences, University of Illinois at Chicago, Chicago, IL USA; 5 Penn State University College of Medicine, Hershey, PA USA; 6 Center for Innovations in Quality Effectiveness and Safety, Houston, Texas USA

**Keywords:** Barriers and facilitators for change, Organizational implementation strategies, Research policy, Research funding

## Abstract

**Background:**

Audit and feedback has been shown to be instrumental in improving quality of care, particularly in outpatient settings. The mental model individuals and organizations hold regarding audit and feedback can moderate its effectiveness, yet this has received limited study in the quality improvement literature. In this study we sought to uncover patterns in mental models of current feedback practices within high- and low-performing healthcare facilities.

**Methods:**

We purposively sampled 16 geographically dispersed VA hospitals based on high and low performance on a set of chronic and preventive care measures. We interviewed up to 4 personnel from each location (*n* = 48) to determine the facility’s receptivity to audit and feedback practices. Interview transcripts were analyzed via content and framework analysis to identify emergent themes.

**Results:**

We found high variability in the mental models of audit and feedback, which we organized into positive and negative themes. We were unable to associate mental models of audit and feedback with clinical performance due to high variance in facility performance over time. Positive mental models exhibit perceived utility of audit and feedback practices in improving performance; whereas, negative mental models did not.

**Conclusions:**

Results speak to the variability of mental models of feedback, highlighting how facilities perceive current audit and feedback practices. Findings are consistent with prior research  in that variability in feedback mental models is associated with lower performance.; Future research should seek to empirically link mental models revealed in this paper to high and low levels of clinical performance.

**Electronic supplementary material:**

The online version of this article (10.1186/s13012-018-0764-3) contains supplementary material, which is available to authorized users.

## Background

The Institute of Medicine (IOM) strongly advocates the use of performance measures as a critical step toward improving quality of care [1, 2]. Part of the mechanism through which performance measures improve quality of care is as a source of feedback for both individual healthcare providers and healthcare organizations [3]. Audit and feedback is particularly suitable in primary care settings, where a higher incidence of chronic conditions is managed, and thus, the same set of tasks is performed multiple times, providing the feedback recipient multiple opportunities to address and change the behavior in question.

However, a recent Cochrane review concluded that audit-and-feedback’s effectiveness is highly variable, depending on factors such as who provides the feedback, the format in which the feedback is provided, and whether goals or action plans are included as part of the feedback [4, 5]. Related audit-and-feedback work recommended a moratorium on trials comparing audit and feedback to usual care, advocating instead for studies that examine mechanisms of action, which may help determine how to optimize feedback for maximum effect [5].

The concept of *mental models* may be an important factor modifying the effect of audit and feedback on organizational behaviors and outcomes. Mental models are cognitive representations of concepts or phenomena. Although individuals can form mental models about any concept or phenomena, all mental models share several characteristics: (a) they are based on a person’s (or group’s) belief of the truth, not necessarily on the truth itself (i.e., mental models of a phenomenon can be inaccurate), (b) mental models are simpler than the phenomenon they represent, as they are often heuristically based, (c) they are composed of knowledge, behaviors, and attitudes, and (d) they are formed from interactions with the environment and other people [6, 7] People may form mental models about any concept or phenomenon through processing information, whether accurately or through the “gist,” see [8, 9]; mental models are thought to form the basis of reasoning and have been shown in other fields of research to influence behavior (e.g., shared mental models in teams positively influences team performance when mental models are accurate and consistent within the team [10, 11]. For example, research outside of healthcare suggests that feedback can enhance gains from training and education programs [12], such that learners form mental models that are more accurate and positive than when feedback is inadequate or not delivered.

However, it is important to note that mental models can manifest at various levels within an organization (i.e., person, team, organization), such as those within a primary care clinic. A facility, hospital, or organization-level mental model represents the beliefs and perceptions of an organization such that these influences are felt by individual members of the organization. Research on clinical practice guideline (CPG) implementation has established empirical links between a hospital’s mental model of guidelines and their subsequent success at guideline implementation: specifically, compared to facilities who struggled with CPG implementation, facilities who were more successful at CPG implementation exhibited a clear, focused mental model of guidelines, and a tendency to use feedback as a source of learning [3]. However, as the aforementioned study was not designed a priori to study audit-and-feedback, it lacked detail regarding the facilities’ mental models about the utility of audit-and-feedback, which could have explained why some facilities were more likely than others to add audit-and-feedback to their arsenal of implementation tools. The present study directly addresses this gap in order to better shed light on the link between feedback and healthcare facility effectiveness.

### Study objective

This study aimed to identify facility-level mental models about the utility of clinical audit and feedback associated with high versus low-performing outpatient facilities (as measured by a set of chronic and preventive outpatient care clinical performance measures).

## Methods

### Design

This research consists of qualitative, content, and framework analyses of telephone interviews with primary care personnel and facility leadership at 16 US Department of Veterans Affairs (VA) Medical Centers, employing a cross-sectional design with purposive, key-informant sampling guided by preliminary analyses of clinical performance data. Methods for this work have been described extensively elsewhere in this journal [13] and are summarized herein. The study protocol was reviewed and approved by the Institutional Review Board at Baylor College of Medicine. We relied on the Consolidated Criteria for Reporting Qualitative Research (COREQ) guidelines for reporting the results herein (see Additional file 1).

### Research team and reflexivity

Interviews were conducted by research staff (Kristen Smitham, master’s level industrial/organizational psychologist, Melissa Knox, registered dietitian; and Richard SoRelle, bachelor’s in sociology) trained specifically for this study by the research investigators (Drs. Sylvia Hysong, PhD and Paul Haidet, MD). Dr. Hysong (female) is an industrial/organizational psychologist; Dr. Haidet (male) is a general internist; both researchers are experienced in conducting, facilitating, and training personnel (see Hysong et al. [13] in this journal for details of interviewer training protocol) and were research investigators at the time the study was conducted. The research team also received additional training on qualitative analysis using Atlas.ti (the analytic software used in this project) specifically tailored for this study from a professional consulting firm specializing in qualitative analysis. The research team had no prior relationship with any of the interviewees prior to study commencement.

### Participants

We interviewed up to four participants (total *n* = 48) at each of 16 geographically dispersed VA Medical Centers, as key informants of the mental models and culture of their respective facilities. Participants were drawn from the following groups: the facility director, the chief of the primary care service, full-time primary care physicians and physician extenders, and full-time primary care nurses. We sought one interviewee per role category and sought to interview clinicians with at least 3 years in their current position to ensure they would have sufficient organizational experience to form more complete mental models. Table 1 summarizes which roles were interviewed at each facility. 5/16 facility directors and 3/16 primary care chiefs declined to participate (75% response rate for facility leaders); securing 24 clinician interviews (12 MD, 12 RN) required 104 invitations [13].

**Table 1:** Site characteristics and roles interviewed at each site

Performance category	Site	Size (# of unique patients)	Residents per 10k patients†	Number of primary care personnel	Interviewee role			
					FD	PCC	MD	RN
High performers	B	27,222	0.00	35	✓	✓	✓	✓
	H	27,851	8.62	62		✓		✓
	*M*	43,845	18.25	56	✓	✓		
	*R*	*49,813*	*31.42*	*83*			✓	
Consistently moderate	*D*	44,022	26.18	115	✓	✓	✓	✓
	E	63,313	10.63	94	✓	✓	✓	✓
	*K*	46,373	56.93	125	✓	✓	✓	
	*P*	*80,022*	*21.45*	*54*		✓		✓
Highly variable	*A*	60,528	23.15	143	✓	✓	✓	✓
	*G*	49,309	26.24	27	✓		✓	✓
	*L*	21,327	7.03	30			✓	✓
	*Q*	*39,820*	*2.89*	*10*	✓	✓		
Low performers	C	44,391	27.51	88	✓	✓	✓	✓
	F	19,609	0.00	46	✓	✓	✓	✓
	J	58,630	24.94	116	✓	✓	✓	✓
	*N*	*24,795*	*0.00*	*23*		✓	✓	✓

Note: sites listed in italic type were sites excluded from the study due to insufficient data (either insufficient number of interviews or insufficient information about mental models was provided by the interviewees of a site during the interviews, thus making any findings from that site unstable). *FD* facility director, *PCC* primary care chief, *MD* physician, *RN* registered nurse. ^†^Number of residents per 10k patients is intended as a measure of the strength of the academic mission of the facility, which has been shown to be a nuanced indicator than the dichotomous medical school affiliation measure used traditional [25]

### Site selection

Sites were selected using a purposive stratified approach based on their scores on a profile of outpatient clinical performance measures from 2007 to 2008 extracted from VA’s External Peer Review Program (EPRP) one of VA’s data sources for monitoring clinical performance used by VA leadership to prioritize the quality areas needing most attention. EPRP is “a random chart abstraction process conducted by an external contractor to audit performance at all VA facilities on numerous quality of care indicators, including those related to compliance with clinical practice guidelines” [14]. The program tracks over 90 measures along multiple domains of value, including quality of care for chronic conditions usually treated in outpatient settings such as diabetes, depression, tobacco use cessation, ischemic heart disease, cardiopulmonary disease, and hypertension.

For site selection, we focused on metrics of chronic and preventative care, as patients may return to the provider for follow-up care (as opposed to an urgent care clinic or emergency department). Table 2 displays the specific measures used to select the 16 sites in the study, which fell into one of four categories: high performers (the four sites with highest average performance across measures), low performers (the four with lowest average performance across measures), consistently moderate performers (the four with moderate average performance and the lowest variability across measures), and highly variable facilities (i.e., the four with moderate average performance and the highest variability across measures). Our study protocol, published earlier in this journal [13], describes the method of calculating the performance categories in greater detail. Table 1 summarizes basic site characteristics grouped by performance category.

**Table 2:** Clinical performance measures employed in site selection

EPRP mnemonic	Short description
c7n	DM-outpatient-foot sensory exam using monofilament
Dmg23	DM-outpatient-HbA1 > 9 or not done (poor control) in the past year
Dmg28	DM-outpatient-BP > =160/100 or not done
Dmg31h	DM-outpatient-retinal exam, timely by disease (HEDIS)
Dmg7n	DM-outpatient-LDL-C < 120
htn10	HTN-outpatient-Dx HTN and BP > = 160/100 or not recorded
htn9	HTN-outpatient-Dx HTN and BP < = 140/90
p1	Immunizations-pneumococcal outpatient-nexus
p22	Immunizations-outpatient-influenza ages 50–64-
p3h	CA-women aged 50–69 screened for breast cancer
p4h	CA-women aged 21–64 screened for cervical cancer in the past 3 years
p6h	CA-patients receiving appropriate colorectal cancer screening (HEDIS)
smg2n	Tobacco-outpatient-used in the past 12 months-nexus-non-MH
smg6	Tobacco-outpatient-intervention-annual-non-MH with referral and counseling
smg7	Tobacco-outpatient-meds offered-nexus-non-MH

Used with permission from Hysong, Teal, Khan, and Haidet [9]

### Procedure

Participants (key informants) were invited to enroll in the study initially by e-mail followed by phone calls if e-mail contact was not successful. Participants were interviewed individually once for 1 h via telephone by a trained research team member at a mutually agreed upon time; interviews were audio-recorded with the participant’s consent (four participants agreed to participate but declined to be audio recorded. In these cases, a second research team member was present to take typed, detailed notes during the interview). Only participants and researchers were present during the interviews. Key informants answered questions about (a) the types of EPRP information the facilities receive, (b) the types of quality/clinical performance information they actively seek out, (c) opinions and attitudes about the utility of EPRP data (with specific emphasis on the role of meeting performance objectives within the facility), (d) how they use the information they receive and/or seek out within the facility, and (e) any additional sources of information or strategies they might use to improve the facility performance (see Additional file 2 for interview guide). The participants sampled were selected as key leaders and stakeholders at the facility, making them ideally suited for responding to facility-level questions and enabling us to make inferences on trends in facility-level mental models. Interviews were conducted between May 2011 and November 2012.

### Clinical performance change over time

**Fig. 1:**
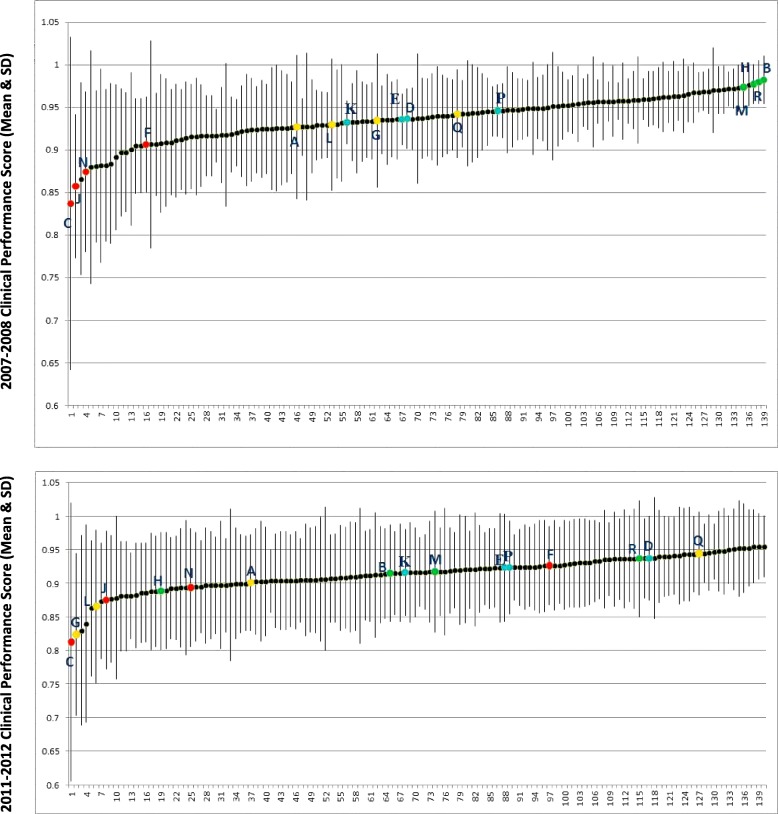
Mean clinical performance scores and standard deviations for all VA Medical Centers in 2007–2008 vs. 2011–2012. Note: colored points represent four performance categories of 16 sites used in this study: red = low, yellow = highly variable, blue = consistently moderate, green = high. In both graphs, the colors represent the category assignments the sites received in 2008 to show the extent to which their relative positions may have changed in 2012

Sites were originally selected based on their performance profile and membership in one of four performance categories. However, the sites did not necessarily remain in their original performance categories throughout the life of the study. Figure 1 presents the average performance scores^1^ of all available medical centers and their respective standard deviations. The colored data points represent the sites selected for the study; the respective colors represent their performance arms as determined for 2007–2008 (see Fig. 1 note). Performance for the rest of the medical centers is depicted by the black data points and is presented purely to show the participating sites’ relative standing. As shown in the upper graph in the figure, the sites clustered cleanly into the four desired arms in 2007–2008. However, as shown in the lower graph, the same sites shifted positions in 2011–2012; for example, site C, which was originally a low performer in 2008, is the third highest performer of the participating sites in 2011–2012, more importantly, the sites are spread throughout the continuum of performance, rather than forming clean, discreet clusters as they did in 2008. Consequently, our original plan to make inferences about the similarities in mental models based on the original performance clusters was no longer viable nor was it possible to re-categorize the sites in 2011–2012 for the same purpose. We therefore adopted an alternate analytic approach to our research question; rather than explain differences in mental models among sites of known clinical performance, we sought to explore differences in clinical performance among sites with similar mental models.

### Data analysis

#### Identifying mental models for each site

Interview recordings were transcribed and analyzed using techniques adapted from framework-based analysis [15] and content analysis [16, 17] using Atlas.ti 6.2 [18]. In the instances where no audio recording was available (*n* = 4), interviewer field notes served as the data source for analysis.

Open coding began with one of the four research team coders reviewing the transcript, identifying and tagging text passages indicative of facilities’ mental models of feedback within the individual interviews using an a priori code list (e.g., positive/negative, concerns of trust, or credibility of feedback) to which they could add emergent codes as needed; coders proceeded with axial coding using a constant comparative approach [13]. For the purposes of coding, we defined mental models as mental states regarding “leaders’ perceptions, thoughts, beliefs, and expectations” of feedback [19]. Coders organized the flagged passages by role and site, then compared the organized passages, looking for common and contrasting topics across roles within a site. Topics across roles were then iteratively synthesized by the research team into a facility-level mental model of feedback derived from the corpus of coding(s), resulting in a 1-page summary per site describing the mental models observed at the facility. A sample site summary and individual responses that led to that site summary are presented in Additional file 3.

Using the 1-page site-level summaries as data sources, we identified common themes across sites, derived from the data; the themes were organized into three emergent dimensions characteristic of the observed mental models (see the “Results” section, below). Coders were blind to the sites’ performance categories throughout the coding and analysis process. After completing the aforementioned analyses, coders were un-blinded to examine the pattern of these mental model characteristics by site and clinical performance cluster.

Data from four sites (one from each clinical performance category) were not usable after initial coding. Reasons for this include insufficient (e.g., a single interview) or unreliable (e.g., interviews from individuals whose tenure was too short to develop a reliable institutional mental model) to infer facility level mental models. Thus, our final dataset comprised 12 sites which were further coded by the lead author in terms of the facility’s mental model positivity, negativity, or mixed perceptions of feedback. Excluded sites appear in gray italics in Table 1.

#### Relating mental models of feedback to clinical performance

As mentioned earlier, due to the change in sites’ performance profiles in the period between site selection and data collection, we sought to identify clusters of sites with similar mental models to explore whether said clusters differed in their clinical performance. To accomplish this, we first sought to identify an organizing framework for the 12 site mental models identified as described in the previous section. The research team identified three dimensions along which the 12 facilities’ emergent mental models could be organized: *sign (*positive vs. negative) perceived feedback *intensity* (the degree to which respondents described receiving more or more detailed feedback in any given instance) and perceived feedback *consistency* (the degree to which respondents described receiving feedback at regular intervals).

With respect to mental model sign, facility mental models were classified as positive if it could be determined or inferred from the mental model that the facility perceived EPRP or other clinical performance data as a useful or valuable source of feedback; they were classified as negative if it could be determined or inferred from the mental model that the facility did not find utility or value in EPRP or similar clinical performance data as a feedback source.

Facility-level mental models were classified as being high in perceived feedback intensity if participants reported receiving more frequent or more detailed performance feedback and were classified as low in perceived feedback intensity if participants discussed the receipt of feedback as being particularly infrequent or not very detailed.

Facility mental models were classified as high in perceived feedback consistency if participants reported receiving feedback at regular, consistent intervals and low in perceived feedback consistency if participants reported believing that they received feedback at irregular intervals.

Once classified along these dimensions, we conducted a logistic regression to test whether categorization among these three levels predicted the sites’ clinical performance categorizations. Separate regressions were conducted for 2008 and 2012 performance categories.

## Results

### Mental models identified

Various mental models emerged across the 12 sites regarding feedback. Therefore, we opted to categorize mental models into either positive or negative mental models of the utility and value of EPRP as a source of clinical performance feedback. This organizing strategy enabled categorization for all but two facilities, which exhibited mixed mental models in terms of sign. Negative mental models focused on the quality and the consequences of the EPRP system and were more prevalent than the positive mental models, which depicted EPRP as a means to an end. We present and describe the types of mental models emergent from the data in the subsequent sections.

### Negative mental models: EPRP does not reflect actual care quality

The mental model observed across the greatest number of sites in the interviews was that EPRP was not an accurate reflection of actual quality of care (and by extension was not a good source of feedback), a theme raised at 6 of the 12 sites coded but displaying the highest levels of groundedness at sites C, K, and M. This mental model was attributable to respondent perceptions that the data were not accurate because of the chart review and sampling approach (e.g., sites C, K, and M, quoted below).

I: Is there anything they could be doing to improve the way you all view EPRP …?

P: Um well uh yeah just being more accurate. I mean the fact that we can um challenge the- the- the reports and that we do have a pretty decent proportion that- that are challenged successfully. … the registries that we’re building I really think are going to be more accurate than either one because there’s a look at our entire panel of patients whether or not they showed up. … it does seem a little backwards these days for someone to be coming through and looking at things by hand especially when it doesn’t seem like they’re being too accurate.

-- Primary Care Chief, Site C

I mean the EPRP pulls from what I understand, I mean sometimes we sorta see that but it’s pretty abstract data. It’s not really about an individual panel. It’s, it’s based on very few patients each month and you know, now I guess it’s pulled at the end of the year but in terms of really accessing that as a provider, um, uh that doesn’t happen much.

-- Physician, Site K

Being able to remove ignorance and make that data available in a user-friendly way is paramount and important and unfortunately from my perspective; our systems and data systems. We are one data-rich organization. We’ve got data out the ying-yang but our ability to effectively analyze it, capture it and roll it out in a meaningful way leaves a lot to be desired;

-- Facility Director, Site M

### Positive mental models: EPRP is a means to an end (transparency, benchmarking, and strategic alignment)

We also observed an almost equally prevalent, positive mental model at a different set of sites (*n* = 4), where interviewees viewed the EPRP as a means to an end (improving quality of care). The specific manifestation of that end varied from site to site. For example, at one site, EPRP was a way to improve transparency:

We try to be totally transparent. Uh sometimes to the point of uh being so transparent people can see everything … and sometimes they may not sound good but if you consistently do it I think you know people understand that.

-- Facility Director, Site B

At another site, EPRP was perceived as a tool for *strategic alignment*:

I think the VA is- is um I think they are wise in connecting what they feel are important clinical indicators with the overall performance measurement and the performance evaluation of the director so that the goals can be aligned from the clinical staff to the administrative staff and we’ve been very fortunate. We’ve gotten a lot of support [latitude in how to best align] here.

-- Primary Care Chief, Site A

At other sites, EPRP was seen as a clear tool for benchmarking purposes:

You benchmark what your model or your goal or your best standards of care are, and that is basically on the spectrum that embodies the whole patient, all the way from the psychological aspect to the community aspect or the social workers too. …That’s the way that I see EPRP. EPRP is only you try to just set up several variables that you can measure that at the end of the day will tell you, you know what, we are taking a holistic approach to this patient and we are achieving what we think is best in order to keep this patient as healthy as possible.

-- Primary Care Chief, Site H

-- Physician, Site L,

### Mixed mental model: EPRP is not accurate, but it helps us improve nonetheless

Of note, site L exhibited an interesting mix. Like the sites with the more negative mental models, they reported concerns with the quality of the data being presented to them. However, this site was unique in that, despite the limitations of the data, respondents at this site nonetheless use the data for benchmarking purposes because, if the targets are met it is an indication to them that a certain minimum level of quality has been reached at the sites.

I’d start worrying and looking at why or what am I doing that’s causing it to be like this. Is it the way they pull the data? Because it’s random. It’s not every single patient that is recorded. Um, they pull, I believe anywhere from 5 to 10 of your charts. … So I do ask that and then if it is a true accounting then I go “OK then it’s me. It’s gotta be me and my team.” I look at what my team is doing or what portion of that, that performance is performed by my staff and what portion of it is by me. And then from there I go OK. Then I weed it out.

### Unintended negative consequences: EPRP is making clinicians hypervigilant

In addition to the mental models described above, a second, negative mental model was observed at a single site about the unintended consequences of feeding back EPRP data—clinicians reported a feeling of hypervigilance as a result of the increased emphasis on the performance measures in the EPRP system:

There’s just a lot of pressure, uh you know, that, uh it seems like leaders are under and you know, I’m in a position where I try to buffer some of that so that my providers don’t feel the pressure but when performance measures and, um other, uh things are being looked at, um and it comes down, uh you know, through our, our electronic medium almost instantly, um it’s, uh you know, looking for responses, why did this happen, it’s very hard for people to feel, feel comfortable about anything.

-- Primary Care Chief, Site F

### Relating mental models of feedback to clinical performance

Table 3 presents the 12 sites, a summary of their respective mental models, and their classification according to the three dimensions in 2008. No significant association was observed between performance (in 2008 or 2012) that can either affect intensity or consistency when tested via logistic regression and analysis of variance with sites as between subject factors, though we acknowledge 12 is a rather small sample size for this type of test. However, we did observe two noteworthy, descriptive patterns: first, none of the sites’ mental models were highly positive; in other words, at best, sites perceived EPRP performance measure-based feedback to be a means to an end (transparency, benchmarking, or strategic alignment), rather than a highly valued, integral best practice for delivering high-quality care. Second, 75% of sites exhibited moderate or low levels of feedback intensity.

**Table 3:** Summary of sites’ mental models and degree of positivity, intensity, and consistency

Performance category (2007–2008)	Site	Performance category (2011–2012)	Mental model summary	Sign/theme	Intensity	Consistency
High performers	B	Moderate	Aim: EPRP feedback is communicated, tracked, and improved upon in a ubiquitous, transparent, non-punitive, systematic, and consistent way.	Positive: transparency	Medium	High
	H	High	EPRP as a benchmark or model for the best standards of care for keeping the whole patient as healthy as possible.	Positive: benchmarking	Medium	Low
	M	Moderately high	EPRP measures are generally OK, but not sophisticated enough to be reflect actual care quality	Negative: EPRP not a good representation of quality	Medium	High
Consistently moderate	*D*	Moderate	EPRP serves as a primary means of linking the work/efforts of all facility staff to the facility’s mission: to provide the best quality of care that veterans expect and deserve	Positive: strategic alignment	Medium	Medium
	K	Moderate	EPRP is not a real true reflection of the quality of one’s practice because of the sample size at a particular time period.	Negative: EPRP not a good representation of quality	Medium	Medium
	E	Moderately high	Clinicians think EPRP is inferior to their population-based, VISN created dashboard, and leaders have concerns about overuse and misinterpretations or misuse of EPRP	Negative: EPRP not a good representation of quality	Medium	Medium
Highly variable	A	Low	EPRP remains relevant as a starting point for setting, aligning, and monitoring clinical performance goals	Positive: strategic alignment	Medium	High
	G	Moderately low	EPRP as an “outside checks and balance” system that validates whether or not how the facility thinks they are doing (e.g., good job) and what challenge areas they have are accurate, however, there are no real or punitive consequences to scoring low.	Mixed	Medium	Medium
	L	Low	Although EPRP does not reflect actual care quality, the numbers indicate that they are doing something consistently right that helps their patients	Mixed	Low	High
Low performers	F	Moderate	Immediate feedback is advantageous to memory, but not always well received.	Negative: EPRP has made us hyper-vigilant	High	Medium
	C	Low	EPRP is viewed by some as an objective, unbiased measure with some sampling limitations; by others, EPRP is viewed as inaccurate or retrospective	Negative: EPRP not a good representation of quality	Medium	Low
	J	Low	Site struggles to provide feedback; clinicians did not receive EPRP and PMs until the PACT implementation.	No feedback until PACT	Low	Medium

Note: 2012 performance categories differ from 2008 because 2012 performance forms a continuum rather than discreet categories (see Fig. 1 and main text for details)

Our final analyses examined descriptively whether sites with positive vs. negative vs. mixed mental models exhibited common patterns of clinical performance. Among sites with negative mental models, all four clinical performance categories from 2008 were represented; clinical performance also varied considerably among these sites in 2012, though no high performers were observed among these sites in 2012. Among sites with positive mental models, the low clinical performance category was the only category not represented in 2008, whereas all levels of clinical performance were represented in 2012. Sites with mixed or neutral mental models exhibited mostly low performance during both time periods (site L, who exhibited highly variable performance in 2008, was the lowest performer in the highly variable category). Of note, sites L and J were also the only two sites with low intensity of feedback.

## Discussion

This study aimed to identify mental models of audit and feedback exhibited by outpatient facilities with varying levels of clinical performance on a set of chronic and preventive care clinical performance measures. Consistent with the highly individualized cultures and modi operandi of individual VA facilities across the country, we found high variability in facilities’ mental models of audit and feedback, and no clear relationship between variability among these facilities’ mental models of audit and feedback and their facility-level clinical performance. Interestingly, one area of consistency across all facilities is that, without prompting, mental models of EPRP reported by interviewees included some component of the quality of the data.

These findings are consistent with previous research noting considerable variability in individual and shared mental models of clinical practice guidelines [3]. Additionally, the finding that the two sites with neutral mental models and by extension, low intensity of feedback exhibited low clinical performance is consistent with previous research indicating feedback intensity may moderate feedback effectiveness [4]. Contrary to previous research, however, no particular mental model of feedback was found to be associated with better clinical performance. Two possible reasons for this include the conditions under which information is processed (e.g., fuzzy trace theory suggests affect impacts information processing and memory recall; [20, 21]), and the local adaptation for feedback purposes of a standardized, national program such as EPRP.

Another possible reason for the lack of relationship between clinical performance and mental models could concern source credibility. The strongest negative mental model indicated providers simply did not perceive EPRP to be a credible source of clinical performance assessment. A recent model of physician acceptance of feedback [22] posits that low feedback source credibility is more likely to be associated with negative emotions such as apathy, irritation, or resentment; however, depending on other factors, this emotional reaction can lead either to no action (no behavior modification) or counterproductive actions (e.g., defending low performance rather than changing it); the model does not specify what factors may compel someone to adopt one pathway (e.g., inaction) vs. another (e.g., action resulting in negative outcomes). Although half of the sites reported having a mental model consistent with low source credibility, our data did not capture each site’s specific pathway from emotional reaction to behavioral outcome and impact. It is therefore possible that unspecified factors outside our data could have been the deciding factor, thus explaining the variability in clinical performance, even within sites with a strong negative mental model of EPRP as a source of feedback.

### Implications

Our study demonstrates the need to extend theory- and evidence-based approaches to feedback design and implementation to foster receptive mental models toward feedback [5]. Implementing effective feedback practices may elicit receptiveness to the feedback systems in place, allowing healthcare organizations to reap greater gains from performance management systems that cost millions to sustain. One noteworthy implication of this study is that positive mental models regarding feedback hinge partially on the feedback system’s ability to deliver clinical performance data in ways the various users can understand quickly and act upon personally (that is, feedback can be translated into an actionable plan for individual improvement [4, 23]). The strongest and most common mental model observed was that EPRP was not a good representation of care quality—if the feedback recipients question the credibility of the feedback, it is unlikely to effectively change performance.

### Limitations

As with all research, this study had limitations. While data were being collected for this study, a seminal quality improvement initiative was implemented: VA’s nationwide transition from traditional primary care clinics to Patient Aligned Care Teams (VA’s implementation of the Primary Care Medical Home model) occurred in 2010. This may in part account for the absence of an observable relationship between mental models and clinical performance. Team-based care involves more complex coordination among clinical staff and involves new roles, responsibilities, and relationships among existing clinical personnel, which may in turn tax clinicians enrolled in the current study [24]. Further, clinicians and divisions of health service may require time to adjust to their new roles, responsibilities, relationships, and changes in workflow. We believe this is illustrated in our data as the mean performance score across all sampled facilities dips significantly from 2008 to 2012 (2008: mean = .935, 95% CI = .004; 2012: mean = .913, 95% CI = .004; *p* < .001), variability in performance across measures is visibly greater for all facilities observed (2008: mean stdev = .05, 95% CI = .004; 2012: mean stdev = .07, 95% CI = .003; *p* < .001), and our sites’ standings in the distribution materially changed (see Fig. 1 for all of these patterns). It is therefore possible this initiative was a sufficiently large system change to possibly overwhelm any potentially detectable relationship between mental models of feedback and clinical performance.

Second, we were unable to use all the data we collected due to the limited number of interviews at some sites (*n* = 1; see data analysis section for more details); however, all four performance categories contained one site with unusable data, thereby minimizing any risk of bias toward a given performance category.

## Conclusions

Despite a national, standardized system of clinical performance measurement and tools for receiving clinical performance information, mental models of clinical performance feedback vary widely across facilities. Currently, evidence supports a strong negative mental model toward feedback likely driven by source credibility concerns, suggesting that EPRP measures may be of limited value as a source of clinical audit and feedback to clinicians. Although our data are at this time inconclusive with respect to the relationship of these mental model differences to performance, the variability in mental models suggests variability in how feedback systems relying on EPRP were implemented across sites; future work should concentrate on identifying implementation factors and practices (such as establishing the credibility of clinical performance measures in the eyes of clinicians) that lead to strong, productive mental models of the innovation being implemented.

## Additional files

Additional file 1: COREQ (COnsolidated criteria for REporting Qualitative research) checklist. (PDF 489 kb)

Additional file 2: Interview guide. (DOCX 26 kb)

Additional file 3: Example site summary. (DOCX 19 kb)

## Footnotes

1 Defined as the average of all clinical performance measure scores used in the performance profile for a given facility in a given performance period [13].
