# Supplementary material for: Mental models of audit and feedback in primary care settings
Source: Implement Sci. 2018 May 30;13:73. doi: 10.1186/s13012-018-0764-3 (PMC5975441; doi:10.1186/s13012-018-0764-3)
Supplement: Supplementary file 2 — Interview guide. (DOCX 26 kb) [file 13012_2018_764_MOESM2_ESM.docx]

**Additional File 2**

**Interview Guide**

# Sample Interview Guide for Leaders

#### Intro / Rapport Building

| Concepts Tapped | Question | Potential Probes |
| --- | --- | --- |
| Warmup/rapport building | 1. Tell me about your role at the VA. |  |
| Progress of PACT rollout at facility | 1. *To what extent has PACT been implemented at your facility? | - How long has PACT been around at your facility? - What does PACT look like right now at your facility? - Have all Primary Care staff members been integrated into PACTs? - Has the facility been able to hire the appropriate amount of staff members to fully staff each PACT? - How are you able to evaluate the progress of the PACT rollout (what data do you receive that provides an effective measure of how far along the PACT rollout is at your facility)? |
| How they personally monitor their own performance -- precursor | 1. In your efforts to ensure delivery of the highest quality primary care to veterans, how do you go about assessing the quality of care that *your Facility* currently provides? | - What do you find valuable about <whatever strategy they just said> - How do you set (or learn about) organizational priorities for your facility regarding clinical performance? - In response to “I know I’m doing well when my patients are happy and healthy,” ask as follow-up: How do you go about finding that out? Or what is it that lets you know they are happy/healthy? |

#### Exploration Area #1: Perceptions of EPRP and Clinical Performance Measurement

| Concepts Tapped | Question | Potential Probes |
| --- | --- | --- |
|  | 1. How is clinical performance measured in your facility? | - What tools are most valuable for gauging (or improving) clinical performance? Why? - * Since the introduction of PACT at your facility, how has the measurement and assessment of clinical performance changed, if at all? - How well does what gets measured match with what is important? |
| EPRP Mental models | 1. When you hear ‘EPRP’, what comes to mind? | - You can preface by saying “People from different facilities may think differently about EPRP; when you hear those letters, what comes to *your* mind? - We know there is a “textbook” definition, we’re interested in your concept of EPRP? - What does EPRP mean for you? - Give me your understanding of what EPRP is all about. - Tell us your understanding of EPRP   - measures   - data collection   - report delivery   - and how the results are used |
| EPRP as feedback | 1. How does EPRP fit in to the measurement of clinical performance at your facility? | - In what ways has EPRP been useful? - In what ways has EPRP not been useful? - If EPRP is one of many sets of critical measures they are responsible for, ask about their, their Facility and/or VISN’s current and past priorities. To what extent are clinicians exposed to EPRP data? To what extent are they exposed to other data related to EPRP measures? - What mechanisms are in place for providers to learn about EPRP measures/data? What education is provided them about EPRP measures/data? - How is the process of EPRP data collection and use explained to clinical staff? |
| Impact of PACT on feedback systems | 1. *Since the transition to PACT, what changes have been made to the clinical performance information made available to your staff? | - How has the introduction of PACT affected the facility’s priorities? - At this time, what performance measures receive the greatest emphasis at your facility? How has this changed over time? - Have you changed the nature of clinical performance feedback reports to physicians/other staff because of PACT? |

#### Exploration Area #2: Feedback Strategies

| Concepts Tapped | Question | Potential Probes |
| --- | --- | --- |
| Feedback characteristics | 1. Tell us about how you give (and receive) feedback about clinical performance at your facility. | - How involved are you in delivering clinical performance feedback to others in the organization? If little to no involvement, to whom does this responsibility get handed off? How are these people held accountable? - By what means is feedback given? What led you to adopt that approach to feedback? - How has this approach been altered due to the implementation of PACT? - How are PACT team members other than physicians given feedback regarding their clinical performance? - What data inform the feedback that is given? - How have PACT measures been incorporated, if at all, into the feedback that is provided to others in the facility? - *To confirm EPRP as only feedback source:* - In what ways other than through EPRP do you provide feedback about clinical performance? |
|  | 1. Tell me about the last time you gave feedback about clinical performance. | - *If their answer does not include distributing EPRP, then ask*: Tell me about the last time you distributed EPRP data. Also, ask: Tell me about the last time you received EPRP data if not addressed in detail previously. - *If this isn’t going anywhere, it might help to focus it a little more*: - Tell me about the last time you delivered feedback about clinical performance that led someone to change their practice. S-B-O^[[1]](#footnote-1)^: What was the situation? What feedback did you deliver? What was changed? - Give me an example of feedback you gave that resulted in little to no change in practice. S-B-O: |
| Feedback acceptance | 1. What do you expect people to do with feedback when you give it to them? (leadership) | - Do you (do your clinics, service line, facilities, etc.) develop an action plan for the feedback? - What means do you have for following up to see if any changes have been made? - *How are the PACT teamlets expected to use clinical performance information? |
|  | 1. What are the consequences of feedback at your facility? *(i.e., what happens if someone is given feedback about clinical performance and chooses to ignore it)* | - What avenues are there if you don’t agree with a piece of feedback you receive? *(e.g., appeal, grievance, is it all just handled informally)* - How are people held accountable to the feedback they receive? |
| Feedback seeking | 1. What could your facility/VISN be doing that they’re not doing now to better track clinical performance? *What could your facility/VISN be doing to better inform PACT teams about their clinical performance? | - What could be done to help clinicians better track their clinical performance? - How would those things help you with your clinical performance? |
|  | 1. Is there anything else that we have not discussed that you would like to share? |  |

1. S-B-O: Situation-Behavior-Outcome [↑](#footnote-ref-1)
