# Supplementary material for: Mental models of audit and feedback in primary care settings
Source: Implement Sci. 2018 May 30;13:73. doi: 10.1186/s13012-018-0764-3 (PMC5975441; doi:10.1186/s13012-018-0764-3)
Supplement: Supplementary file 3 — Example site summary. (DOCX 19 kb) [file 13012_2018_764_MOESM3_ESM.docx]

**Additional File 3**

**Example Site Summary**

**Sample Site– Mental Model: EPRP “EPRP is viewed by some as an objective, unbiased measure with some sampling limitations; by others, EPRP is viewed as inaccurate or retrospective.”**

# FD: EPRP is an objective, externally compiled tool that shows the facility’s clinical performance.

- AACOS: EPRP has been used inaccurately over the past year or so years – a new discovery.
- MD: EPRP does not exist here; clinical performance feedback is minimal, retrospective, and punitive.
- RN: EPRP is used here; however, feedback is punitive.

# FD: EPRP is an objective, externally compiled tool that shows the facility’s clinical performance.

The Facility Director states that historically (past 5+ years) they performed poorly. Over the past 18-36 months, (when s/he and the Chief of Staff arrived), their performance numbers went from not living up to the national VA performance standards to one that exceeds them. (FD 051). The FD primarily believes EPRP is a useful source of facility-level data (not provider-level) (FD 167-179, FD 071). S/he states EPRP is objective and gathered externally, thus unbiased. (FD: 077-083) S/he does however state that EPRP’s small sample size and measure definitions sometimes causes the VA to lose sight of the goal (i.e., taking care of the patient), thus consuming a lot of the staff’s energy. (FD 133-137, FD 141) In regards to how EPRP and the PACT fit together, FD thinks that PACT consists of smaller teams providing the same care as previously done thus EPRP and the like are still relevant under the PACT model. (FD 203)

FD states that now providers receive more panel specific performance data from the AACOS; thus, s/he thinks that providers have actionable data (FD 199) and very good data that show them how they stack up against their peers. (FD 195). The FD also believes that since EPRP is always talked about, providers are aware of EPRP. (FD-181-183, FD 187). If providers have a grievance about CP FB/evaluations, the FD states their approach to FB is very positive, but if somebody wants to object, there’s not much to argue given their data based-approach. (FD 355-361)

**AACOS: EPRP has been used inaccurately over the past year or so years – a new discovery**

The Acting ACOS stated that s/he doesn’t want to be in this role nor know much about his/her duties, but s/he is trying to catch on. (ACOS 081-085). S/he notes that their numbers last time were not good. S/he explains that he is trying to determine how to introduce EPRP to the PCPs. Previous ACOSs simply provided clinical reminder (CR) reports without mentioning EPRP. (ACOS 155-179). S/he tries to stress that CRs (98% of the time) are reflections of good care. (ACOS 230) S/he explains that the facility’s response to EPRP had been to run the clinical reminders report to double check the EPRP numbers; so, unless the CR reports show similar results to EPRP, they will not change what they are doing. (ACOS 256).

However, s/he states that the first thing that comes to mind when s/he hears EPRP is “inaccurate” due to the small number of charts reviewed as well as a discovery made by the new AO, that is, there are some glitches in their clinical reminder reports. They have been using 2009 data instead of more recent year either 2011 or 2012. S/he notes that these numbers are used in part to determine P4P at year end. (ACOS 099). Thus, reports are being rewritten now.

When asked how the clinical performance numbers impact this role, s/he states “it’s really hard for me to tell honestly. I’m definitely not in the Pentad’s head. S/he mentions that s/he and another service chief (behavioral health) had to respond to “bad numbers” at the last QPWG meeting. Thus, s/he feels like it’s his responsibility as ACS Chief, but s/he doesn’t have a full picture of how the numbers impact him yet (ACOS 181-203) and that no one has ever explained the purpose or for what measures s/he is responsible. (ACOS 220).

**MD: EPRP does not exist here; clinical performance feedback is minimal, retrospective, and punitive.**

The MD favors a proactive feedback environment. S/he is familiar with EPRP from prior military experience but s/he is not aware of EPRP at the current facility. S/he receives “HEDIS” data. The clinical performance FB s/he receives is minimal, retrospective, and reactive; s/he only receives FB about the things related to bonuses. (MD line 183). Peer reviews don’t provide honest FB due to fear of retribution. FB culture seems punitive. ACOS is acting and supervisor is a former resident (10 yrs. Junior to the interviewee) and doesn’t provide clinical performance FB.

**RN: EPRP is used here; however, feedback is punitive.**

Although the RN is aware of EPRP and says EPRP relates to clinical reminders, review of a larger scale than just local, accountability being held for everyone equally (RN 395-407), s/he knows that she does a good job with clinical reminders (CRs) and other tasks they are assigned because it’s policy and Ambulatory Care mandates that certain behaviors be performed and things done. (RN 027). S/he knows that CRs are “supposed to reflect good medical care”, but at the facility it is used more as a tool to reward people and give them bonuses. (RN 041). S/he hadn’t received a performance evaluation since XXXX until last summer; the participant went to the union and demanded an evaluation. (RN 2:30) S/he also describes the RN staff meetings led by the new/current nurse manager as punitive and unsupportive (e.g., “brow beaten every month”. (RN 2:40)

**FD (6/6/2012)**

**AACOS (4/19/2012)**

**MD (05/04/2011)**

**RN (9/26/2011)**

**Turnover:** Interviewee (Interview date: April 2012) has been the Acting ACOS since August 2011 (ACOS 013) and had worked at the facility for a couple of years prior to getting this “stupid acting job”. (ACOS 077). The ACOS before them was also “acting” from January 2010 to July 2011.

ACOS states that they’ve had almost entire turnover of important admin staff in Ambulatory Care and both the nurses and the physicians are feeling pretty low and angry. (ACOS 222). RN explains that about four years ago, around 20 physicians left along with some nurses. (RN 137-147)
